# Co-bedding as a Comfort measure For Twins undergoing painful procedures (CComForT Trial)

**DOI:** 10.1186/1471-2431-9-76

**Published:** 2009-12-11

**Authors:** Marsha L Campbell-Yeo, C Celeste Johnston, KS Joseph, Nancy L Feeley, Christine T Chambers, Keith J Barrington

**Affiliations:** 1 Women's and Newborn Health Program, IWK Health Centre, Halifax, Nova Scotia, Canada; 2 McGill University, Montreal, Quebec, Canada; 3 The Perinatal Epidemiology Research Unit, Departments of Obstetrics & Gynecology and Pediatrics, IWK Health Centre and Dalhousie University, Halifax, Nova Scotia, Canada; 4 Centre for Nursing Research, Jewish General Hospital and The Quebec Interuniversity Nursing Intervention Research Group (GRIISIQ), Montreal, Quebec, Canada; 5 Centre for Pediatric Pain Research and the Departments of Pediatrics and Psychology, IWK Health Centre and Dalhousie University, Halifax, Nova Scotia, Canada; 6 Division of Neonatology Sainte-Justine University Hospital and Université du Montréal, Montréal, Quebec, Canada

## Abstract

**Background:**

Co-bedding, a developmental care strategy, is the practice of caring for diaper clad twins in one incubator (versus separating and caring for each infant in separate incubators), thus creating the opportunity for skin-to-skin contact and touch between the twins. In studies of mothers and their infants, maternal skin-to-skin contact has been shown to decrease procedural pain response according to both behavioral and physiological indicators in very preterm neonates. It is uncertain if this comfort is derived solely from maternal presence or from stabilization of regulatory processes from direct skin contact. The intent of this study is to compare the comfort effect of co-bedding (between twin infants who are co-bedding and those who are not) on infant pain response and physiologic stability during a tissue breaking procedure (heelstick).

**Methods/Design:**

Medically stable preterm twin infants admitted to the Neonatal Intensive Care Unit will be randomly assigned to a co-bedding group or a standard care group. Pain response will be measured by physiological and videotaped facial reaction using the Premature Infant Pain Profile scale (PIPP). Recovery from the tissue breaking procedure will be determined by the length of time for heart rate and oxygen saturation to return to baseline. Sixty four sets of twins (n = 128) will be recruited into the study. Analysis and inference will be based on the intention-to-treat principle.

**Discussion:**

If twin contact while co-bedding is determined to have a comforting effect for painful procedures, then changes in current neonatal care practices to include co-bedding may be an inexpensive, non invasive method to help maintain physiologic stability and decrease the long term psychological impact of procedural pain in this high risk population. Knowledge obtained from this study will also add to existing theoretical models with respect to the exact mechanism of comfort through touch.

**Trial registration:**

NCT00917631

## Background

An increasing number of multiple pregnancies and increasing obstetric intervention at preterm gestation has led to a rising number of preterm twin infants being admitted into Neonatal Intensive Care Units (NICU). These preterm infants undergo repeated and often untreated procedural pain that can contribute to immediate stress and may have a long term impact on the normal maturation of regulatory systems. The practice of co-bedding twins simulates various aspects of the intrauterine environment. Co-bedding allows twins to remain in close proximity and have skin-to-skin contact with each other, thus creating opportunity for familiar recognition of auditory and olfactory stimuli and for a continuation of the twin relationship that began in utero.

Given the potential benefits of co-bedding, theoretical and conceptual underpinnings, and compelling evidence related to the effects of environmental context, it is important to examine the possibility that twins placed in close proximity could provide comfort and protection against the numerous stressful procedural assaults experienced during hospitalization. The intent of this study is to compare the comfort effect of co-bedding (contrasting twin infants who are co-bedding versus those who are not) on pain response during a tissue breaking procedure (heelstick). Pain response will be measured in all infants and will be determined by physiological and behavioral reaction.

## Summary of Literature Review

### a. Recent increase in twin births and preterm births among twins

During the past two decades, significant advances in medical technology have contributed to the increased survival of critically ill, preterm, and very low birth weight infants [1]. As mortality rates have declined, the focus has shifted to decreasing morbidity and adverse neurodevelopmental outcomes for these high-risk infants [2]. An additional change in the surviving population within NICU's is that the numbers of twins admitted has escalated as the occurrence of multiple births is continuing to rise in North America [3]. In Canada, multiple births increased from 2.1 per 100 total births in 1991 to 2.7 per 100 total births in 2000, [4] and to 3.0 per 100 total births in 2004 [5]. Advanced maternal age and increased fertility treatments have been reported as the main reasons for this increase [3,6-8]. These factors have been primarily associated with a rise in dizygotic twins. Although race has some effect on the on the incidence of dizygotic twinning (10-40 per 1000 live births among people of African descent compared to 7-10 per 1000 among people of European descent), higher maternal age and assisted reproductive technologies are strongly associated with multiple gestation [9]. Occurrence of monozygotic twinning has been less affected with a stable incidence of 4 per 1000 total births worldwide.

Preterm birth is the leading cause for hospitalization during the neonatal period and is responsible for more than 75% of all cases of perinatal morbidity and mortality [10]. The incidence of preterm birth among multiples has risen substantially over the past several decades in North America. In the United States, preterm birth rates among twins increased by 19.6%, from 40.9% in 1981 to 55% in 1997 [11] and to 60.5% in 2005 [12]. In Canada, the rate of preterm birth among twin live births increased by 14.5%, from 42.5% between 1985 and 1987 to 49.6% between 1994 and 1996 [13], to 53.0% in 2000 [4] and 57% in 2004 [14]. Furthermore, twin pregnancies that followed assisted reproduction were more likely to result in lower mean gestational age (33.1 versus 34.2 weeks) and mean birth weight (2,029 versus 2,177 g for the first twin and 1,897 versus 2,136 g for the second twin) than those occurring spontaneously, with longer associated NICU stays [15]. In a similar study in 2002, examining the effect of multiple births on perinatal indicators over two decades in Canada, the British Isles, France and the Unites States, twins contributed to a disproportionate share of preterm deliveries and low birth weight newborns [16]. Maternal and neonatal complications associated with twin pregnancies also contribute to the increased likelihood of NICU admission and prolonged hospitalization. Excess maternal risks in twin pregnancies include gestational hypertension, placental abruption, and placenta previa, all which are positively correlated with adverse neonatal sequellae [17]. In a 2005 Canadian review of 3,242 infants born at or before 32 weeks of gestational age and admitted to 24 Canadian NICUs, twins had approximately similar mortality when gestational age and severity of illness were accounted for (adjusted odds ratio 1.3, 95% confidence interval 1.0-1.6) [18]. Weight discordance, chorionicity, and gestational age at birth were more closely associated with adverse outcomes than plurality of pregnancy [19]. However, the higher likelihood of these factors in twin versus singleton birth created the additional morbidity risk associated with multiple births [20].

### b. Ubiguitous pain exposure in the neonatal intensive care unit

Given the higher likelihood of preterm birth and associated morbidity leading to the need for NICU admission, twin infants often face increased medical challenges and can be neurodevelopmentally less prepared to cope with multiple stimuli after birth when compared to healthy full-term infants [21]. Data from several countries have consistently shown that neonates undergo multiple painful and stressful procedures during hospitalization in the NICU. Scandinavian [22] and British [23] studies report an average of 10-15 procedures daily with younger neonates (< 28 weeks) undergoing as many 700 painful procedures during their hospital stay [24]. Recently, Carbajal [25] conducted a 2-week prospective chart review of 431 infants in 13 European NICUs. These infants endured 60, 969 painful procedures and a mean of 16 exposures (range 0 to 62) of painful or stressful procedures per day. Pain management for infants undergoing procedural pain associated with most frequent procedures such as tracheal suctioning, heelstick, tape removal, venepuncture and intravenous line insertions, although improved in recent years, was suboptimal. Almost 40% of infants undergoing heelstick for blood collections, the most commonly performed tissue breaking procedure in the NICU setting, did not receive any form of non-pharmacologic or pharmacologic intervention and 41% of infants underwent tracheal intubation without the benefit of any pain relieving strategies [25]. In a similar 1-week prospective chart review of pain practices in 14 tertiary level NICU's in Canada, Johnston and colleagues [26] reported that 582 infants underwent a total of 30,416 procedures. Thirty-five different procedures were identified with 3553 (11.7%) being classified as tissue breaking (i.e. skin-breaking or endotracheal intubation or an ophthalmologic examination). On average infants had 26 exposures (0-469) per week, just less that 4 per day. Although these findings support a slight reduction in previously reported average number of daily painful procedures that infants in the NICU endure, pain relieving strategies were still not routinely used. Forty-six percent (tissue) and 57% (non-tissue) damaging procedures performed were not accompanied by any form of pain relieving treatment.

Animal studies have linked pain to adverse developmental changes in the brain [24,27] and in the spinal dorsal horn [28,29]. In human infants, immediate responses to untreated pain such as physiological elevations in heart rate, blood pressure, and oxygen requirements, can lead to fluctuations in intracranial pressure, possibly leading to intraventricular hemorrhage (IVH) and periventricular leukomalacia [30,31]. Increased stress hormone release triggered by pain impedes normal regulation of growth and tissue repair [32] and has adverse effects on cognition, memory, and behaviour systems [33]. Stress associated with pain can lead to prolonged structural and functional alteration in pain pathways that lasts into adult life, permanently altering normal or common responses to pain [34,35]. Given the extreme plasticity of the preterm brain and immature regulatory processes, it is not surprising that exposure to repeated skin breaking procedural pain may disrupt the normal development of physiological, hormonal, behavioural and hypothalamic-pituitary-adrenal (HPA) axis that may contribute to these long term effects. Recently, Grunau and colleagues have reported a blunting of hypothalamic-pituitary-adrenal (HPA) axis response in infants who had undergone numerous painful procedures in the NICU [29,36]. Preterm infants in contrast to infants born at term [37,38] appear to experience a down-regulation of behavioural responses and a decrease in sympathetic recovery contributing to higher physiological instability.

Despite a surge in the literature illustrating various methods to accurately assess and manage pain and the provision of consensus practice guidelines [39] minimal improvement in the treatment of pain associated with routine NICU procedures has ensued. The reasons for this lack of practice change are unclear. Issues related to research utilization (i.e. education, unit context and institutional facilitation) and lack of consensus regarding optimal pain management strategies for routine procedural pain are the most likely cause. In addition, evidence that morphine (a commonly used neonatal analgesic) which is known to attenuate postoperative and severe pain, is less effective for pain associated with mechanical ventilation and heelstick [40] as well as possible adverse outcomes associated with its prolonged use [41] has led to further inquiry regarding the role of non-pharmacologic measures and environmental context in the minimization of acute pain.

### c. Environmental context and comfort for alleviation of pain

Infants have been shown to have cortical perception [42,43] and memory of pain, both exhibited by peripheral hypersensitization [37] and behavioural response [29,44,45]. Recently, two studies using near infrared spectroscopy (NIRS) to measure pain experience in preterm infants, revealed that infants as young as 28 weeks of gestation exhibit cortical response during heelstick [42,43]. Functional MRI imaging of adults has demonstrated that pain perception and inhibitory mediation appears to involve multiple areas of the brain, referred to as the "pain matrix" [46], and that perception and response can be mediated by visual cues and relational factors [47]. Although not yet proven with neonatal neuroimaging, the assumption that neonates may also perceive and respond to pain and distress in a similar interlinked manner is highly plausible. It is known that pain in newborns can be soothed with alterations in environmental context and provision of non-pharmacological interventions involving orogustatory, vestibulokinesthetic, and/or olfactory and tactile systems. Sweet tasting solutions, breastfeeding and nonnutritive sucking regulated through endogenous opiate and serotonin systems have been shown to diminish pain response associated with procedural pain [48-51]. Containment, felt to enhance regulation of infant state via swaddling and facilitated tucking have also been shown to be beneficial [49]. Although the benefits of music and vestibular action may be less promising in isolation (i.e., without the mother), these results have helped us better understand the importance of maternal presence and relationship with respect to pain response [52,53].

Both term and preterm infants have olfactory memory. They not only show preference for their own mother's amniotic fluid and breastmilk, but this recognition has been shown to diminish crying during maternal separation and pain response during heelstick [54-57]. Interestingly, olfactory recognition of a familiarized smell can illicit a similar comforting response [58,59] indicating both memory and ability to learn, remember and have emotional connections even in young, very preterm infants. Kangaroo mother care (KMC) or skin-to-skin care (SSC) provides a multisensorial context encompassing tactile, olfactory, and relational systems. It has been shown to diminish pain response and improve physiological stability in both term and preterm infants [60-66]. Whether the mother is an essential aspect of this comfort during skin-to-skin contact has yet to be proven.

The discovery and practice of new and innovative approaches to minimize the effects of infant pain should be a primary focus of neonatal health care researchers [67]. Numerous possible non-pharmacologic measures or alternative environmental contexts within the NICU have yet to be fully explored as primary or adjunctive methods to relieve pain and diminish potential long lasting effects of pain on the development of regulatory pathways. The increased incidence of multiple gestation births and admission of these fragile babies to neonatal units also raises questions regarding the differences in care of twins and higher order multiples versus singletons. Despite the ever increasing numbers of at-risk twin infants, specific interventions targeted at this population have not been studied.

### d. Co-bedding as a potential comfort measure

At birth, preterm twins are typically separated as individual health needs are met. This leads to an interruption in their shared uterine environment and disrupts the expected developmental trajectory of a twin pregnancy. Co-bedding of twins is an example of a developmental care initiative. Its purpose is to minimize neurodevelopmental sequellae associated with admission to a NICU [21,68,69]. The practice of co-bedding is based on the premise that extrauterine adaptation of preterm twins is enhanced by continued physical contact with the other twin rather than sudden deprivation of such stimuli [69,70]. Maintaining this presence may assist twins to cope with pain associated with routine procedural pain by stabilizing self regulatory pathways.

In summary, twins, the majority of whom are born preterm, are exposed to painful procedures as part of their essential medical care. The adverse effects are both immediate and potentially long-term, affecting future sensation and behaviour [27,34,71]. Given that the practice of co-bedding simulates numerous aspects of environmental context - proximity, tactile, olfactory, auditory, memory and relationship - that have been shown to provide comfort to newborns, it is reasonable to propose that the contact or presence of a twin who has shared the same uterine space since conception would have a similar comforting effect.

## Purpose

The purpose of this study is to determine the efficacy of twin comfort during a tissue breaking procedure (heelstick) in the NICU.

## Hypotheses

The pain response of a twin undergoing a heelstick will be significantly altered when co-bedding.

## Methods/Design

### a. Study Objectives

The intent of this study is to evaluate the effect of co-bedding among at-risk twins on pain response during a painful procedure. Secondary objectives include the effect of twin co-bedding on changes in physiologic stability and recovery following the painful procedure, heart rate variability, salivary cortisol, frequency of dosages of 24% sucrose given during painful procedures and the response of the twin not receiving the tissue breaking procedure.

### b. Study design

We propose to carry out a randomized controlled trial comparing the comforting effect of co-bedding on twins undergoing a tissue breaking procedure in the NICU.

### c. Study population

The source population consists of all twin pairs admitted to a tertiary level NICU at the IWK Health Centre, Halifax, Nova Scotia and St. Justine Hospital, Montreal, Quebec, who are considered medically stable and who require at least one medically indicated heelstick for blood procurement.

### d. Inclusion criteria

All medically stable twin infants admitted to the NICU of the IWK Health Centre and St. Justine Hospital will be eligible for the study. Twins will be deemed to be medically stable if they are:

a. Free from infection, and

b. Breathing room air or receiving oxygen via nasal prongs.

Twins may be receiving feeds via gavage tubes, IV therapy via peripheral or central line, and may be experiencing periods of apnea.

Additionally, parents of the twins must understand written and spoken English or French.

### e. Selection criteria for study subjects

The parent (s) of twin pairs who meet the inclusion criteria will be approached by the study investigator/nurse and informed of the study. Signed written informed consent will be obtained before recruitment into the study.

### f. Exclusion criteria

Twin infants to be excluded are those who at the time of study entry:

i. Weigh less than 1000 grams;

ii. Are receiving mechanical ventilator support;

iii. Have chest tubes or umbilical catheter in situ;

iv. Have major congenital anomalies or chromosomal aberrations;

v. Only one of the twin pair requires overhead phototherapy;

### g. Allocation of participants to the trial groups

see Figure 1

**Figure 1:**
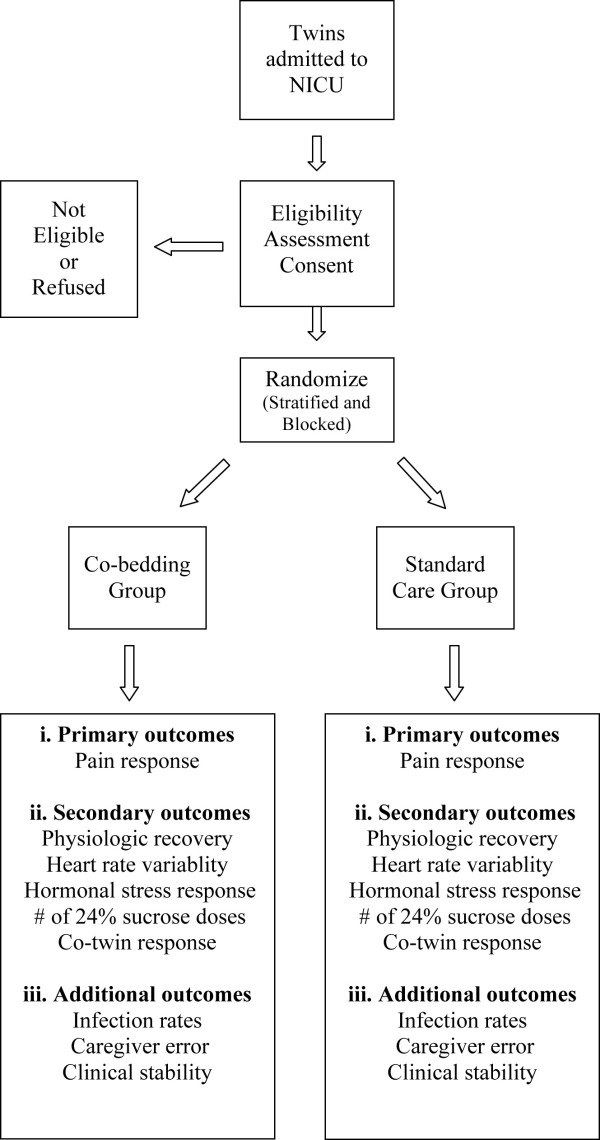
**Allocation of participants to study groups**.

### h. Randomization

Eligible infants whose parent(s) have provided consent will be randomized by a computerized website accessed by the principal investigator or research nurse. Allocation concealment will be ensured using randomly permuted blocks of two, four or six. Infants less than or equal to 31 6/7 weeks will be randomized separately from those twins greater than or equal to 32 weeks. Infants from different study sites will also be randomized separately to ensure identical proportions within the co-bedding and standard care groups.

## What are the proposed methods of protecting against sources of bias?

Due to the unblinded nature of the intervention, it is important to strictly adhere to rigorous methods to eliminate potential bias. The use of an off-site computerized website for group allocation will decrease the risk of allocation bias. The individuals coding pain response will be blinded to the twins treatment status (whether co-bedding or receiving standard care). The video camera will be set up to focus only on the infant's face and will be controlled by the investigator or research nurse. Coding of pain scores will be calculated using the Premature Infant Pain Profile (PIPP) [72]. The PIPP is considered a reliable and valid tool to measure procedural pain in both preterm and term neonates [73-75]. Both composite PIPP scores and combined behavioral (facial) indicators will be reported in each group. Coding will be carried out at McGill University at Dr. Johnston's lab by 2 coders (coder A and coder B). Each coder will only code data on infants who are co-bedding (A) or code data on infants who are receiving standard care (B). Coders will not enter the unit, communicate with each other or compare data sets (i.e. PIPP scores will be estimated without knowledge of group assignment). The use of different coders (who have no previous knowledge of patient enrolment and randomization to code separately for infant facial responses, etc and who will remain blinded to each other's assessments), is expected to minimize observer bias. Research coders A and B will be trained separately by the principal investigator and a member of Dr. Johnston's research lab team. Training will be standarized and coding performances assessed so that an interclass coeffecient (ICC) of 0.85 is reached between the coders' standarized scores. Following the initial training, coders will be retested quarterly using standarized videotapes to ensure inter-rater reliability. If the ICC drops below 0.75, re-training and re-coding will take place. This reliability check conducted with standarized tools will minimize the likelihood of observer bias. Every three months, coders A & B will also re-code two randomly selected videotapes from the first weeks of coding to ensure intra-rater reliability. ICC's of 0.75 will be considered the cutoff point of acceptability. Following study completion, previously assigned group coders will be asked to code a random selection of approximately 20% (n = 6) of the tapes from the alternate group. Inter-rater scores will be correlated to ensure that if differences are found between the groups, these differences are related to the intervention of co-bedding and not to systematic error between the two coders scoring techniques.

In addition, every attempt will be made to assign the same nurse to care for a set of twin pairs regardless of which group they have been assigned. Adherence to this aspect of the study protocol will be recorded daily until the study heelstick has been completed.

## What is the intervention and proposed duration of treatment?

### a. Co-bedding care

Following randomization to the co-bedding group, twin infants will be placed together in a Giraffe Incubator or crib lying side-by-side. Twins will be diaper clad and nested together in boundaries consistent with neonatal care practices. Larger infants may be partially clothed if in an open crib but still able to freely touch one another and remain nested together. Twins will be positioned close to each other (lying face-to-face, back-to-back, or in spooning positions), permitting contact between them. All infants will have cardio-respiratory monitoring while co-bedding. One side of the incubator/crib will be for twin 'a' and one side will be for twin 'b' and infants and their equipment will be colour coded. Incubator temperatures will be determined using clinical reasoning, anticipated neutral thermal environmental needs (based on infant weight, gestational and post natal age) and incubator settings prior to initiation of co-bedding. If servo temperature regulation is required, most likely in the case of younger twin pairs or discordance in infant weights, the servo probe will be placed on the larger infant. Infant temperatures will be closely monitored and recorded throughout the co-bedding condition to maintain normal axilla readings between 36.8 and 37.2 degrees Centigrade.

All infants will be co-bedded for no less than 24 hours prior to heelstick to allow for stabilization following transfer. The heelstick being studied will occur no greater than 10 days following initiation of co-bedding. Duration of co-bedding will be recorded and controlled for in the analysis if necessary. Limiting the length of co-bedding duration decreases the degree of variance possible for this variable yet still allows adequate time for a heelstick to be ordered as part of usual care.

The medically indicated heelstick will be performed by a designated group of experienced nurses and lab technicians who have performed heelstick procedures in previous studies in the NICU in a standardized manner according to the institutional and unit policy. The nurse assigned to care for the twin will assist with the heelstick procedure. Their role will be to provide non-pharmacologic measures as per the NICU pain guidelines as they would do normally. None of the members of the research team or nurses conducting heelstick will prompt the bedside nurse to alter their care in any way. All non-pharmacologic strategies for pain relief implemented by the nurse including number of 24% sucrose doses given will be recorded and confirmed with video recorded data.

Infants assigned to the co-bedding group, may continue co-bedding, should their parents choose, up to 48 hours prior to discharge at which time monitors will be disconnected and the infants separated.

### b. Standard care

For infants who are randomized to receive standard care, the twin pair will remain in separate incubators as per current NICU policy. The twins will be nested in boundaries consistent with neonatal care practices. The heelstick may occur at any time following randomization but within 10 days to maintain consistency between groups. Twins will undergo a medically indicated heelstick in the incubator or crib in an identical fashion as outlined above.

## What is the proposed frequency and duration of follow-up?

Data will be collected simultaneously on both the infant undergoing the heelstick and his/her twin. Data collection using monitoring (Somté and Massimo oxygen saturation systems) and video-tape recording will take approximately 20 minutes per participant - a baseline period (1-2 minutes prior to heelstick), warming (2-3 minutes), heelstick (2-5 minutes), and recovery phase (approximately 1-10 minutes).

If both infants require a medically indicated heelstick on the same day, they will occur no less than 30 minutes apart.

All chart data will be collected following randomization and data collection will continue until completion of the heelstick. Prior painful procedures will include all procedures from birth until completion of heelstick.

## What are the proposed outcome measures?

### a. Primary outcome

The pain response to heelstick determined by physiological and behavioral reactions to a painful event (Premature Infant Pain Profile (PIPP) scores) will be compared between co-bedding and standard care groups.

### b. Secondary outcomes

The physiological recovery in response to heelstick determined by the length of time for heart rate and oxygen saturation to return to normal (baseline), heart rate variability, hormonal stress response, frequency of 24% sucrose administration, and the response of the twin not receiving the painful procedure when his/her twin undergoes a heelstick procedure will be compared between co-bedding and standard care groups.

### c. Other outcomes

Clinical stability (incidence of apnea or bradycardia, and need for supplemental oxygen prior, during and following (i.e. recovery period) heelstick, the number of painful procedures experienced by the neonate prior to the heelstick procedure, infection rates, and caregiver error will be compared between the groups.

The measurement of the main variables of the study relies on three strategies: video recording of facial actions, monitoring of cardio-respiratory measures and oxygen saturations, collection of salivary cortisol and chart review (Table 1).

**Table 1:** Key variables, measures proposed, data sources, and time of administration

Variables	Measures	Method/Sources	Time of administration
Pain response	Premature Infant Pain Profile (PIPP)	Videotape of facial responses	Baseline, warming heelstick and recovery

Physiologic recovery	Heart rate, Oxygen saturation	Somté software	Baseline, warming heelstick and recovery

Heart rate variability	Cardiac monitoring	Somté software	Baseline, warming heelstick and recovery

Hormonal stress response	Cortisol	Sorbette oral swab	Prior to heelstick (basal) and 20 minutes after (stress) the heel stick

Frequency of 24% sucrose administration	Count	Chart medication record, confirmed with videotape recording	2 minute prior and during intervention

Co-twin Response	All measures identical to the twin undergoing heelstick, except sucrose count	Identical measures, except sucrose count	Baseline, warming heelstick and recovery

Safety surveillance	Caregiver error Infection rate	Institutional adverse event and infection control surveillance	Quarterly

Clinical Stability	Supplemental oxygen Incidence of apnea Bradycardia	Chart review	Baseline, warming heelstick and recovery

## Sample Size

Previous studies examining the effect of maternal contact or the effect of sucrose on pain response during heelstick have revealed a greater than 2 point difference in PIPP scores [48,53,61,66]. In those studies the intervention (i.e. maternal skin-to skin contact or sucrose administration) were compared to a usual care group that received no form of pain relieving intervention. As practice guidelines now indicate, it is anticipated that all twins regardless of group assignment will receive 24% sucrose prior to undergoing heelstick. Since it is known that sucrose has a large effect on pain response, the intervention of co-bedding will be considered an additional comfort measure. A one-point additional decease in PIPP scores would therefore be considered clinically significant.

We based our PIPP score assumptions on previous studies which reported PIPP scores of 10.7 (2.3) vs. 12.9 (2.5) from a study on kangaroo skin-to skin care versus incubator care in preterm infants 32-36 weeks of gestational age [61] and PIPP scores 8.9 (*CI *7.9-9.9) versus 10.7 (*CI *9.6-11.8) in preterm infant 28-31 6/7 weeks of gestational age. Based on these reported 0.5 and 0.6 standard deviation pain scores [66] and the reported values in the above studies, we used a conservative standard deviation estimate of 2.0 as our proposed study population will encompass both groups of infants. Sample size was calculated using a 2-sided alpha error of 0.05 and a power of 80 percent. We designed the study to detect a difference of 1 point or greater change (SD 2.0) in the PIPP scores. One hundred and twenty-eight infants would be required to identify this variation in the PIPP scores if such a difference is in fact caused by co-bedding. With this sample size, we will also have over 95% power to detect a greater than 15 second mean difference in physiological recovery (heart rate and oxygen saturation) between groups. In a recent study of skin-to-skin care [66], the time to return to baseline heart rate following the application of the plaster signifying the end of the procedure was significantly different, 123 seconds (*CI *103-142) for the Kangaroo Mother Care condition and 193 seconds for Incubator condition (*CI *158-227, *p *< .00001). Since all infants will receive 24% sucrose, we do not expect that the differences seen in time to recovery will be this large. Therefore, by using the larger sample, and conservatively accounting for the use of regression techniques, our study will recruit 128 participants (64 sets of twins, 32 assigned to the co-bedding group and 32 to the standard group).

## What is the planned recruitment rate?

The IWK Health Centre NICU admits on average 70 sets of twins per year. Ste. Justine Hospital has a similar admission rate, averaging 80 sets per year. Recruitment rates for similar trials previously conducted in these units have ranged from 55-80%. Given a conservative recruitment rate of 60%, and expected later start date at Ste. Justine Hospital (within 6 months of initial enrolment at the IWK Health Centre), anticipated length of time for recruitment for the study is 12-15 months.

## Will compliance be a concern?

We have conducted a previous pilot study [76] and are conducting a larger clinical trial examining twin regulation during co-bedding over an extended period of time and have not encountered issues related to compliance. Given the short duration of data collection (during one heelstick procedure), we do not anticipate problems with compliance. Nevertheless, we will monitor compliance to group allocation through daily observation and direct staff communication.

## What is the proposed type and frequency of analysis?

Analysis and inference will be based on the intention-to-treat principle. Efforts will be made to ensure that follow up is complete for all subjects and that there are no missing values for any of the subjects for any variable. Blinding of independent coders will be retained until after the analysis is completed. Baseline characteristics of study subjects will be contrasted to ascertain that randomization has in fact produced comparable groups with respect to all variables that effect pain response and physiologic stability. The primary outcome of interest will be the pain response of the infant experiencing a tissue breaking procedure while co-bedding with his/her twin when compared to a twin infant experiencing a tissue breaking procedure receiving standard care (alone in incubator or crib). This analysis will compare the means in the two groups before and after treatment and contrast the mean difference between groups using 95 percent confidence intervals and a p value. The stratified nature of the randomization will be accounted for in the analysis. Also, since twin pairs will be randomized together (i.e. to co-bedding or standard care), the analysis will be corrected for potential non independence of outcomes between twin pairs This will involve appropriate variance adjustment which will be carried out using Generalized Estimating Equations (GEE) procedures using SAS software (Proc Genmod, SAS 8.2, SAS Institute Inc. Cary, NC) [77]. If differences are noted in baseline characteristics, inferences will be made based on observed and (linear regression) adjusted differences between groups. Analysis for secondary outcomes will be done in an identical fashion as pain response and recovery by comparing the mean changes in the two groups before and after treatment and contrasting the mean differences between groups using 95 percent confidence intervals and a p value.

## Trial management

The principal investigator will assume the role of study coordinator as part of the requirements of her PhD studies. She will be responsible for the day-to-day management of the trial at the IWK Health Centre. An experienced research nurse will assume the role of coordinator and responsibilities at the IWK during any absences by the principal investigator. A research nurse, having weekly contact with the principal investigator, will coordinate and oversee data collection at the Ste. Justine site.

## Expected contributions

A number of clinical and theoretical contributions are expected from this study. First, it is anticipated that the results from this study will contribute to evidence-based research in the field of pain in preterm neonate health, specifically as it relates to co-bedding and developmentally sensitive care practices. If twin contact while co-bedding is determined to have a positive effect then changes in current neonatal care practices to include co-bedding for twins may be an inexpensive, non-invasive method to minimize preterm twin infant pain during painful procedures. Additionally, the role of a twin in the care of his co-twin may be clarified with this study. As it was stressed in the review of literature, very little is known about the short or long term contribution that a twin can make through their continued presence with their co-twin during hospitalization. Utilizing the practice of co-bedding as an intervention to reduce pain in their preterm newborn can add to our understanding of the potential strength of the relationship between twins. Additionally, given the increased incidence of multiple gestation births and admission of these high risk infants to neonatal units, if co-bedding is found to be beneficial it may raise further questions regarding the need for possible differences in care of twins and higher order multiples versus singletons in the NICU setting.

Theoretically, this area of research is important because there is an increasing realization that alleviation of pain among infants is critical for healthy growth and long-term development. This research will clarify the role of co-bedding and provide at-risk infants with comfort that will facilitate their optimal growth and development. Data will provide valuable information to help better understand the mechanisms contributing to increased comfort within a multisensorial context. Lastly, the results of this study will provide valuable insight into the relationship between twins - whether they are able to provide comfort to each other. Data from the response of the co-twin as an exploratory question will also address this relationship.

## Potential risks to the safety of participants involved in the study

### a. Co-bedding condition

All twins will have continuous cardio-respiratory monitoring and ongoing surveillance for any adverse effects. If a co-bedded infant shows clinical signs of sepsis, twins will be separated until sepsis has cleared. If the incidence of co-infection among co-bedded twins increases significantly above the unit norm, the trial will be discontinued.

### b. Confidentiality

Confidentiality of all data collected will be maintained. All information gathered would be coded before analysis and data will be stored in a secure, locked location accessible only to the principal investigator and research nurse. The list of code numbers and names will be stored separately from the coded data. When the study results are published or presented at a health care conference, the information shared will not contain any personal identifiers. The salivary cortisol samples, coded with a number will be kept frozen in a locked freezer located at the IWK Health Centre and St. Justine NICU until it is couriered in batches for analysis at the McGill University laboratory. Cortisol samples will not be used for any other purpose. All videotapes will be encrypted. Master copies of research data will be kept secure in a locked location until five years past the age of majority of the infants.

## Role of each investigator

MCY will be responsible for the progress and timely completion of the trial. MCY and KB will be responsible for responding to clinical queries, encouraging recruitment, protocol compliance and accurate and complete data collection. NF and KB will provide advice regarding neonatal issues. CC will be responsible to help with queries regarding pain assessment and coding. KJ will be responsible for providing advice on methodological issues and assisting with interpreting statistical analysis. CJ will provide supervision throughout the study. Study investigators will be obliged to participate in study committee meetings related to study progress and completion.

## Ethics

Authorization and informed consent will be obtained from a parent(s) of eligible twins prior to study entry. The IWK Health Centre, Ste. Justine Hospital and McGill University Research Ethics Board have approved this study. The principal investigator or site research nurse will explain the research study to the parents if twins meet the study criteria. Parents will read the Consent/Authorization Form and any questions they may have concerning the research study will be answered. The Consent/Authorization form will contain information on potential risks and benefits to the participants, research rights of the participant, and information on how to contact the invetigator or study nurse. Participation in the study will be voluntary. Parents will be made aware of their right to withdraw their children at any point in the course of the study. Consent forms will be provided in English or French and a copy will be provided to participants once signed.

Co-bedding is considered to be a safe practice for twins. Nevertheless, a careful watch will be kept on all study participates with regard to any possible adverse effects of co-bedding. Infants will be monitored as per the NICU Care Unit standard of care and their clinical condition will be evaluated daily as part of medical rounds and by the study team. Heelstick procedures are an aspect of usual care for infants in the NICU and will not be conducted solely for the purpose of this study. Study participation will not interfere with routine care practices. Routine strategies for pain relief including sucrose administration and non-pharmacologic measures will be provided as per standard IWK Health Centre NICU care.

Co-bedding twins is not considered to be a standard of care in the NICU. This study provides no direct benefit for the parents or infants enrolled. Compensation will not be offered. There will be no study restrictions regarding the continued practice of co-bedding until 48 hours prior discharge for those infants allocated to the co-bedding group

## Study timeline

The initial preparation time for this study will be two weeks. During that time, information will be given to the staff of the NICU regarding study protocol. A second site (Ste. Justine) will commence recruitment within 6 months of initiation in the first site (IWK Health Centre). Subject accrual and data collection will extend over 12-15 months. It will take an additional 4 months to analyze the data and prepare a manuscript. The study results will be widely disseminated through conference proceedings and peer reviewed publication. The total duration of the study is expected to be 20-24 months.

## Discussion

All staff and parents will be informed that we are co-bedding twins while in the NICU for the purposes of research only. We do not intend for this research to indicate support of co-bedding after discharge. Since there is currently little research completed to support the benefits or risks of co-bedding, this will remain a parental decision. We will recommend that parents follow the back-to-sleep program and refrain from smoking regardless of which ever sleep arrangements they choose.

## Dissemination Plan

The design and scientific merit of this study will provide data that will be applicable to like populations within Canada and elsewhere. The generation of further knowledge in this area will help in the formulation of evidence-based recommendations for vulnerable infants experiencing procedural pain and the role of developmental care practices. This program of research has significant implications for the health of hospitalized at-risk infants, both short and long-term. Ongoing research is aimed at identifying optimal developmental strategies that may lessen the adverse effects of procedural pain. Findings will be communicated locally to front line care providers from multiple disciplines, families and administrators to facilitate uptake of knowledge and generate policy change directly affecting patient outcomes. Changes in practice and outcomes will be communicated via best practice networks to stimulate uptake nationally. The research findings also have worldwide relevance in the area of neonatal care and will be communicated nationally and internationally in peer-reviewed journals, conference proceedings and via consultation with community interest groups (Parents of multiple birth association (POMBA) and related networks (worldwide twin neonatal group-Vermont Oxford Network).

## Competing interests

The authors declare that they have no competing interests.

## Authors' contributions

All authors contributed to the development of the protocol, and read and approved the final manuscript.

## Pre-publication history

The pre-publication history for this paper can be accessed here:

http://www.biomedcentral.com/1471-2431/9/76/prepub
